# Supplementary material for: The interactome of intact mitochondria by cross-linking mass spectrometry provides evidence for coexisting respiratory supercomplexes
Source: Mol Cell Proteomics. 2017 Dec 8;17(2):216–32. doi: 10.1074/mcp.RA117.000470 (PMC5795388; doi:10.1074/mcp.RA117.000470)
Supplement: Supplemental Data [file supp_RA117.000470_133922_0_supp_23978_fzffwf.zip › spectra_annotation/mito_DR_spectra_annotation/18-1-1-1-4-1.pdf]

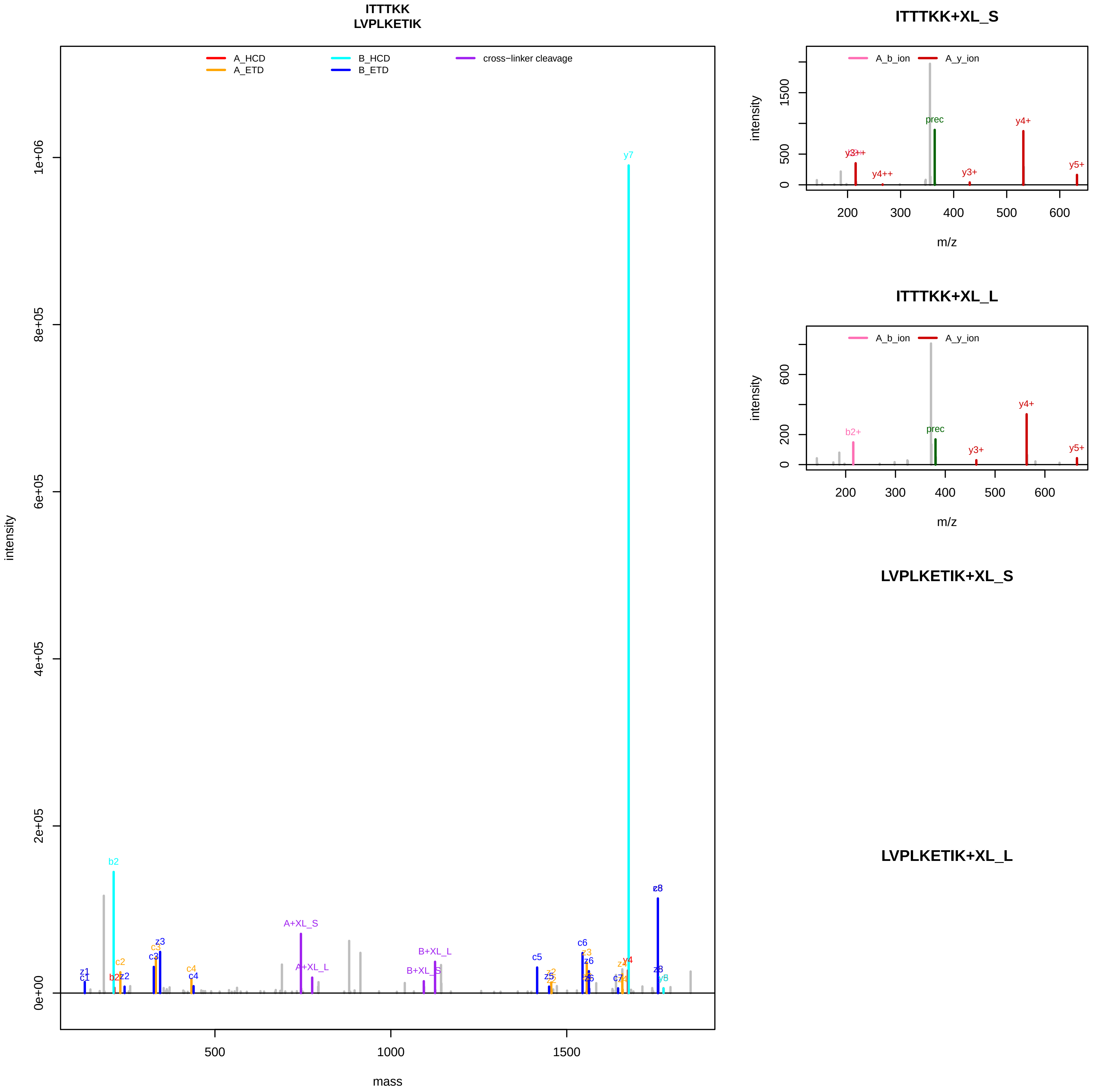
ITTTKK
LVPLKETIK
A_HCD
A_ETD
B_HCD
B_ETD
cross−linker cleavage
intensity
mass
0e+00
2e+05
4e+05
6e+05
8e+05
1e+06
500
1000
1500
y7
b2
z8
A+XL_S
A+XL_L
B+XL_L
B+XL_S
c5
c6
z3
c3
c2
c4
z1
c1
b2
z2
z5
z6
c7
y4
y8
ITTTKK+XL_S
A_b_ion
A_y_ion
prec
y4+
y3++
y4++
y3+
y5+
m/z
ITTTKK+XL_L
b2+
LVPLKETIK+XL_S
LVPLKETIK+XL_L
